# Preparation and sports performance of multilayer flexible anti stabbing fabric martial arts sports composite conjugate materials

**DOI:** 10.3389/fchem.2023.1256541

**Published:** 2023-11-17

**Authors:** Jie Zhang, Huanxiang Ding, Yanqun Wang

**Affiliations:** College of Physical Education and Health, Linyi University, Linyi, Shandong, China

**Keywords:** gas permeability, moisture penetrability, preparation of composite conjugated materials, martial arts sports, multi-layer flexible anti stab fabrics, ultra-high molecular weight polyethylene

## Abstract

Puncture-resistant clothing plays an important role in martial arts. This article studies the preparation process of multi-layer flexible stab-resistant fabrics, analyzes the sports properties of the fabric, and explores the potential application of stab-resistant clothing in martial arts. This article uses ultra-high molecular weight polyethylene fiber as raw material, preprocesses it, including fiber cleaning, drying, lamination, and laminating them together through needle punching, then soaks the laminated fibers in resin, and the soaked fibers solidifies to form a specific flexible puncture-resistant fabric. This article prepares three types of layered fabrics, namely woven fabrics, nonwoven materials, and composite materials of nonwoven materials + woven fabrics + nonwoven materials, and analyzes the kinematic characteristics of the three layered fabrics. Experimental results show that when the number of layers is 4, the average breaking tensile forces of woven fabrics, nonwoven materials and composite materials are 3400, 4600 and 3860 respectively, and the average breaking elongations are 11.8%, 40.6% and 17.4% respectively. This shows that woven fabrics have the highest levels of air permeability and moisture permeability.

## 1 Introduction

With the rapid development of the economy and the integration of global cultures, sports are a combination of entertainment, fitness, and culture (Robert, 2021; Zhang, 2022). The development of sports provides diverse elements for people’s lives. Athletes need to perform specific movements during the exercise process, which can exercise their bodies and provide passionate competitive competition. When performing specific movements, athletes need to wear specific clothing to protect them or provide their athletic abilities. Martial arts is a highly competitive but dangerous sport, and after many years of development, there are more and more types of martial arts. For example, fencing is a highly ornamental sport, but during the process, athletes face the danger of sharp objects piercing them. Fencing requires wearing anti stab fabrics to ensure safety. Anti stab clothing has a huge market for martial arts sportswear, but the sports performance of ordinary anti stab clothing is not good. Ordinary anti stab clothing is often heavy and has poor breathability, leading to martial arts athletes not being agile enough. Conjugated materials can be designed to have excellent puncture and scratch resistance, effectively reducing the risk of injury for athletes in martial arts competitions. The preparation technology of composite materials is constantly developing, and multi-layer flexible anti stab fabrics can retain both the mechanical properties and anti stab properties of clothing through the needle punching process. The application potential of multi-layer flexible anti stab fabrics in martial arts is enormous, and it is of great significance to study the preparation and sports performance of multi-layer flexible anti stab fabrics in the field of martial arts. By preparing multi-layer flexible stab-resistant fabrics with high performance and applying them to martial arts, the safety of martial arts can be improved while maintaining high sports performance.

## 2 Related work

Multi layer flexible anti stab fabric materials have excellent performance, and their application in sports can effectively protect the safety of athletes. Many people have conducted research on the application of multi-layer flexible anti stab fabric materials. Sitotaw Dereje Berihun mixed wool fibers and aramid fibers for 3D (three-dimensional) printing, forming a carbon fiber structure and applying it to the sports field. The constructed textile material has excellent anti stab performance and high comfort. The application of multi-layer flexible anti stab fabric can ensure that athletes are not injured by sharp devices (Sitotaw et al., 2021). Nayak Rajkishore developed specific soft armor through multi-layer flexible anti stab fabric and developed a vest that can resist both pistol bullets and knife stab attacks. Multi layer flexible anti stab fabrics are very different from traditional aramid fabrics, and they also have high softness (Nayak et al., 2018). Li Ting-Ting proposed a flexible polyurethane foam composite with sandwich structure. The sandwich is composed of concave convex fabric structure, and the top/bottom layer is made of polyurethane foam. The Rectangular cuboid is firmly fixed between two layers of low melting point polyester fabric by hot melting. The spacer fabric combination and the number of layers can improve the dynamic cushioning performance (Nayak et al., 2018). An Chao’s research pointed out that individual protection issues are increasingly receiving attention in the new era. He analyzed the motion performance of low-speed impact resistant protective materials based on the applicable range and use of the materials. Most flexible anti stab clothing is first composed of high-performance fibers and resin composite into a single layer of fabric, and flexible anti stab clothing has high wear resistance (An et al., 2019). The development of multi-layer flexible anti stab fabric materials can reduce the risk of injury for athletes during sports, but the materials used in anti stab fabrics can greatly limit people’s activity performance. Traditional martial arts clothing materials are usually made of cotton, silk, hemp and other natural fibers. These materials have limited performance in stabbing and piercing, and cannot provide sufficient protection. Moreover, traditional materials are easy to wear, especially in fierce martial arts sports, and clothing is easy to be damaged and needs to be replaced frequently.

Martial arts is a sport that requires a greater degree of physical activity. Martial arts sports have a high demand for clothing materials, and there are many researches on clothing materials for martial arts sports. Qiu Zhicheng prepared a polyester carbon black system with a carbon black content of 2%–3% using *in-situ* continuous polymerization method, and characterized the rheological behavior, crystallization behavior, and carbon black dispersion morphology of the *in-situ* polymerized polyester/carbon black system. He prepared polymeric polyester/carbon black filaments through high-speed spinning, but the breathability of the polymeric polyester/carbon black filament material is poor (Qiu et al., 2021). Wei Hongqiu summarized the realization methods and material properties of four-dimensional printing Shape-memory polymer, and used various soft polymers as basic materials to study the basic mechanism of shape conversion. Composite elastomer materials have a broad application prospect in sports, biomedical, robot and other fields (Hongqiu et al., 2017). Shalaby Mohammed Nader’s research pointed out that research and development activities in advanced material engineering and nanotechnology have enormous potential, which can completely transform intelligent nano textiles. In the field of sports, the application of flexible anti stab textiles can improve athletic performance while protecting athlete safety (Shalaby et al., 2020). Kamal Mohamed S studied the influence of some structural factors of Sportswear double-layer knitted fabric on the resistance to ultraviolet radiation. Some sports need to be carried out outdoors for a long time. Knitwear is popular with sportsmen because of its flexibility and good wear resistance. By adjusting the structure of textiles, it can achieve a good anti ultraviolet effect (Kamal et al., 2020). It has been proven that martial arts requires high performance of textile materials, and martial arts clothing needs to have anti stab function. However, there is still a lack of relevant research on the analysis of the sports performance of martial arts anti stab textiles.

With the rise of global sports culture, there is a great demand for flexible anti stab fabrics in martial arts. This article prepared multi-layer flexible anti stab fabrics using ultra-high molecular weight polyethylene fibers. The layered structure used woven fabrics, non-woven materials, composite materials, and analyzed the motion performance of multi-layer flexible anti stab fabrics using mechanical testing and subjective evaluation methods.

## 3 Preparation method of multi-layer flexible anti stab fabric

### 3.1 Material selection and combination

Sports are beneficial for people’s physical health and promote cultural integration. Sports competitions follow the laws of human growth and development and physical activity, enriching people’s cultural life. After long-term development, the types of sports have become diverse, and martial arts is an important form of sports.

Martial arts is a tradition of ancient military warfare, where people exercise their bodies by practicing martial arts (Rios et al., 2018; Thomas and Thomas, 2018). Many martial arts events involve sharp tools such as knives and swords, and martial arts require special attention to anti stabs. For example, in fencing competitions, athletes earn points by stabbing their opponents with swords. The visual effect of the fencing competition is stunning, but it poses a certain safety threat to the athletes.

Wushu includes all kinds of movements, such as boxing, legs, hands, feet, elbows, knees and other skills, and requires materials to allow free body movement without restrictions. The material must have high stab resistance and can effectively resist stab and puncture. It also needs to have high strength and durability, and must be flexible enough to allow free body movement without restricting martial arts movements.

Flexible anti stab materials achieve the goal of softness and anti stab by laminating one or more materials and using high-performance fiber structures (Li et al., 2020; Guo et al., 2021). Ordinary flexible anti stab materials usually use woven or non-woven fabrics, but due to their simple structure, their anti stab performance is not stable.

Polyethylene fiber has some advantages, such as high strength, light weight and stab resistance, which makes it one of the popular materials for preparing stab-resistant materials. Polyethylene fiber has excellent strength, which means that it can effectively resist stab and puncture. Compared with some other high-strength fibers, polyethylene fibers are relatively lightweight, which helps to maintain the lightweight of multi-layer stab-resistant fabrics and improve the comfort and flexibility of wearers. Polyethylene fiber usually has excellent durability, which can resist the friction and stress in repeated use, thus prolonging the life of materials.

Polyethylene fiber has lower density, so it is lighter than many other fibers. It also has good strength and wear resistance, so that it can bear certain pressure and tension in various applications. This makes it widely used in textile, packaging, rope, rope net and other durable products.

In order to ensure that the flexible anti stab material not only has anti stab function but also excellent sports performance, this article selects ultra-high molecular weight polyethylene as the raw material. Ultra high molecular weight polyethylene is a polymer compound with very stable chemical properties and excellent wear resistance (Gupta et al., 2018; Wang et al., 2020). Polyethylene fiber is made of high density polyethylene or low density polyethylene, and its molecular structure is linear, consisting of carbon and hydrogen atoms. This linear structure makes polyethylene fiber have high toughness and strength, which is helpful to resist impact force (Xiao et al., 2019). Ultra high molecular weight polyethylene can be well applied in the preparation of anti stab fabric materials for martial arts. The performance parameters of high-strength polyethylene fibers are shown in Table 1.

**TABLE 1:** Performance parameters of high-strength polyethylene fibers.

Serial number	Performance	Parameter
1	Length	51 mm
2	Size	3.3D
3	Strength	28 cN/tex
4	Breaking elongation	2.39%

In Table 1, the performance parameters of high-strength polyethylene fibers are described, with a total of 4 properties. The fiber size of high-strength polyethylene fibers is 3.3D, and the elongation at break is 2.39%.

Due to the different layer structures of fiber raw materials, the overall performance of anti stab materials may vary. Therefore, this article combines three types of layer structures of ultra-high molecular weight polyethylene. The combination methods of the layer structure are: woven fabric, non-woven material, composite material of non-woven material + woven fabric + non-woven material. The design specification of the three ply structure combinations are shown in Table 2.

**TABLE 2:** Design specification of three paving structure combinations.

Layered structure	Woven fabric	Non-woven material	Composite material
Areal density (kg/m^2^)	0.1	0.1	0.1
Thickness (mm)	1.4	3.2	2.2

In Table 2, the design specification of three ply structure combinations are described. The raw materials used are ultra-high molecular weight polyethylene, and the surface density is 0.1 kg/m^2^. The thickness of woven fabric structure is 1.4 mm.

### 3.2 Preparation process flow

Non-woven fabric is a type of fiber polymer that is typically combed into a web of high-performance fibers through needle punching, and anti stab materials are made by repeatedly piercing the web.

The density and structure of non-woven fabrics can effectively reduce the risk of puncture and protect the wearer. Non-woven fabrics are usually more flexible than some hard materials, which is very important for allowing free body movement, especially in martial arts.

When using a needle machine for fiber mesh puncture, it is necessary to use the needle hooks at the edges of the needle machine to drive the surface fibers to move vertically in the direction of the fiber mesh. When a large amount of fibers are penetrated into the fiber mesh, it can play a fixed role. Woven fabrics can be well applied in the field of anti stab, using a plain weave structure with strong anti extrusion ability.

**FIGURE 1:**
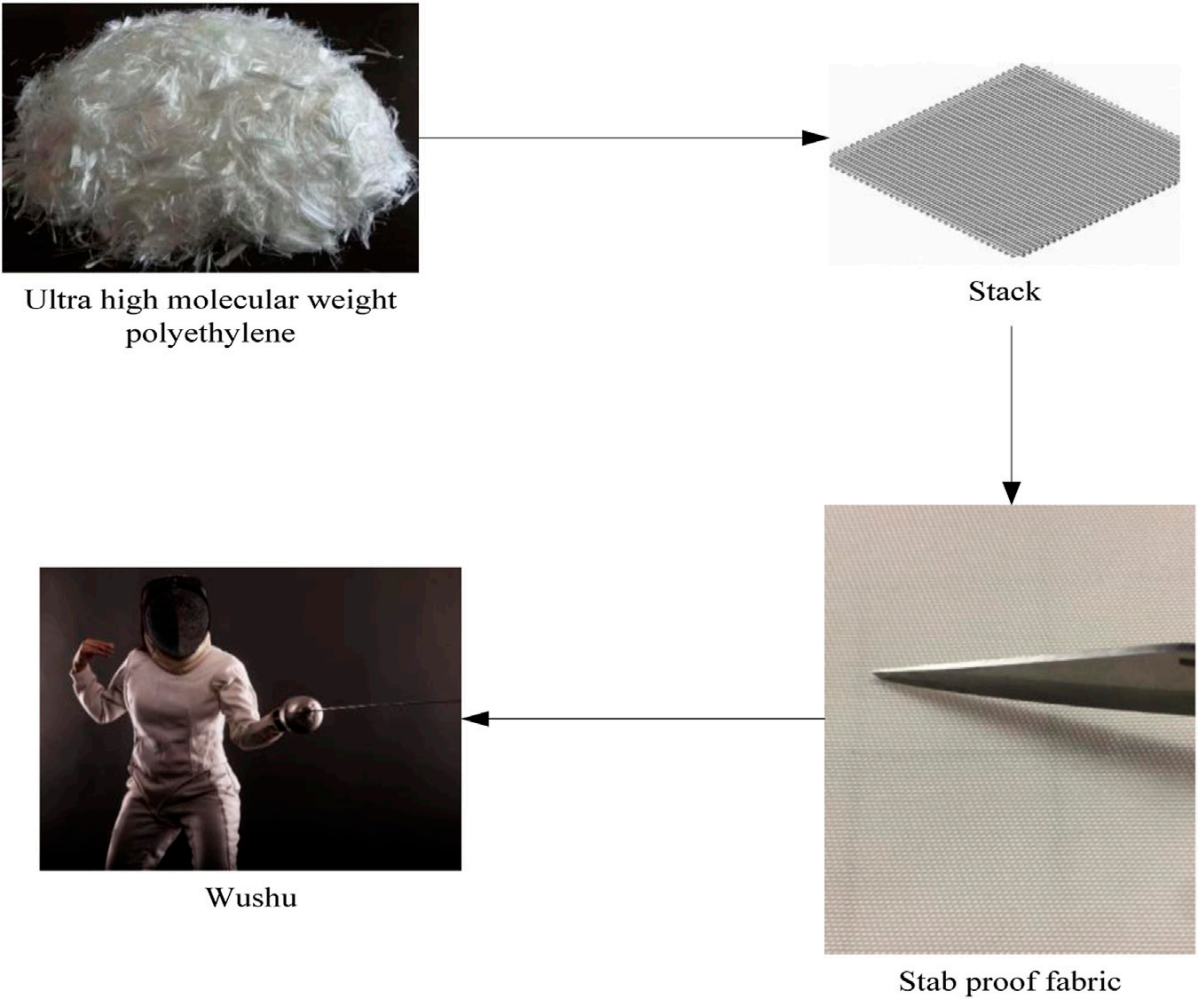
Application process of multi-layer flexible anti stab fabric.

The application process of multi-layer flexible anti stab fabric is shown in Figure 1.

In Figure 1, the application process of multi-layer flexible anti stab fabric is described. By stacking ultra-high molecular weight polyethylene, a textile with anti stab performance can be formed, which can be applied to martial arts to ensure the safety of athletes. The used UHMWPE fiber has an areal density of 0.1 kg/m^2^ and a thickness of 2.2 mm.

**FIGURE 2:**
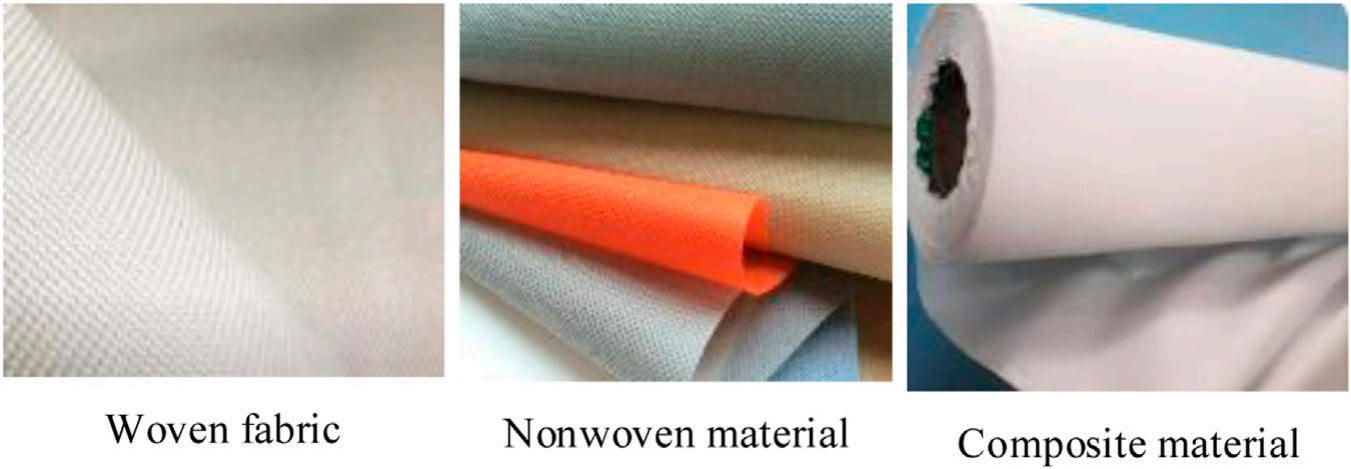
Comparison of different materials.

The comparison of different materials is shown in Figure 2.

In Figure 2, the woven fabric is composed of two groups of cross-woven yarns, which makes the woven fabric have obvious textile patterns, such as plaids or stripes. Non-woven fabrics are made by bonding fibers or fiber sheets, hot pressing or needling, and fibers have no obvious weaving structure in the material. Composite materials adhere non-woven layers to one or both sides of woven fabrics to obtain new materials that combine the characteristics of the two materials.

The preparation process of multi-layer flexible anti stab fabric includes four steps, namely, pretreatment, layering, soaking, and curing. Before processing fiber raw materials, it is necessary to pre-treat the fiber materials. Fiber material pre-treatment involves cutting, cleaning, and drying the fibers to ensure the dryness and quality of the fiber raw materials. After preprocessing, it is necessary to layer the fiber materials in a certain order and number of layers to adapt to different martial arts application scenarios. By fusing the stacked fibers with resin, each fiber gap penetrates into the resin, resulting in a certain thickness and strength of the fibers, ensuring the overall toughness of the fibers. Polyurethane resin is usually used for coating before fusion. Polyurethane is a synthetic resin with excellent strength, elasticity and wear resistance. The application of polyurethane resin in coating can increase the stab resistance and tear resistance of fabric, and also provide a certain degree of waterproof. Finally, the soaked fibers are placed into a fixed mold, which is customized according to the needs of martial arts sports textiles and can form the required size and shape under certain pressure and temperature environments.

After the mold is formed, subsequent processing of the fiber material, including polishing, repair, etc., is required to enable multi-layer flexible anti stab fabrics to adapt to specific scenarios of martial arts.

For high strength polyethylene fiber composites, the preparation temperature is usually below the melting point of polyethylene fiber to avoid the melting and degradation of fiber. The temperature range is controlled between 100°C and 150 C. When preparing composite materials, it is necessary to apply a certain pressure to ensure that the fiber and resin fully contact and form a uniform composite structure, and the pressure ranges from 3 to 5 MPa. The duration of preparing the composite depends on the curing time of the resin and the thickness of the composite, and the duration is half an hour.

The silylated graphene oxide in polymer thermoplastic matrix is a composite material, which combines thermoplastic polymer matrix and chemically modified graphene oxide. This composite material has many physical properties and can be used in different fields in martial arts (Ganguly et al., 2019).

### 3.3 Needle punching process for layering

The key to the preparation of multi-layer flexible anti stab fabrics is fiber stacking, and the anti stab performance is related to the characteristics of the fibers themselves and the structure of the fabric. In order to effectively layer the fibers, the needle punching method is used for material preparation and the performance of the material is adjusted by controlling the parameters of the needle punching to meet the application of different martial arts sports scenarios.

The surface density of the single layer anti stab layer is set to 0.4 kg/m2, and the two layers of non-woven materials and woven fabrics are laminated. The anti stab material is made by driving the vertical crossing of fibers through acupuncture. In order to facilitate the analysis of the material properties of different layer structures, a single-layer anti stab layer is vertically cross laid to make a two-layer non-woven material. The single-layer woven fabric is vertically laid to make a three-layer woven fabric material.

During the lamination process using the needle punching method, due to the extremely fine nature of high-strength polyethylene fibers, they are prone to sticking together. Therefore, appropriate loosening of the fibers is necessary before needle punching. Due to the small gap between fibers, static electricity is generated during the carding process, making it difficult for fibers to stack regularly. Therefore, it is necessary to remove static electricity during the carding process of fibers.

In order to make the fiber carding process easy to control, a digital carding machine is used for carding, allowing high-strength polyethylene fibers to run between two types of card clothing.

The acupuncture machine drives the vertical movement of fibers through acupuncture, thereby achieving fiber stacking. The acupuncture machine can achieve fiber stacking regulation by adjusting feeding parameters, needle plate parameters, and output parameters. The acupuncture process is mainly controlled by the feeding and output speed of the fiber mesh, acupuncture density, and acupuncture frequency.

The needle density is expressed as:

D=M100×S (1)

In Formula (1), D represents the density of needles, needle density refers to the number of needles received per unit area, and S represents the distance each needle machine advances.

The distance that the needle machine advances each time can be expressed as:

S=wm×100 (2)

In Formula (2), w represents the fiber mesh output speed.

Combining Formula (1) and Formula (2), it can be obtained:

D=M×m10000×w (3)

By controlling the acupuncture parameters of the acupuncture machine, precise preparation of multi-layer flexible anti stab fabrics can be achieved, achieving the preparation of anti stab fabrics for different martial arts sports.

**FIGURE 3:**
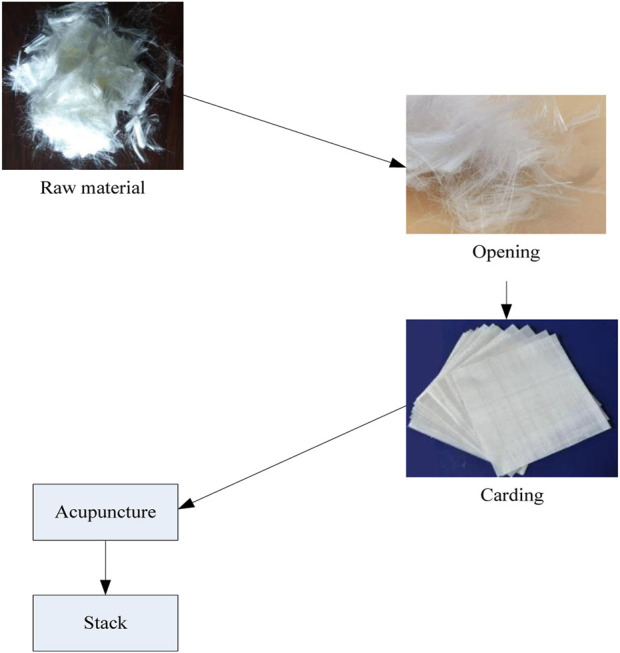
Stacking process of needle punching process.

The process of multi-layer flexible anti stab fabric layering through the needle punching process is shown in Figure 3.

In Figure 3, the process of lamination using the needle punching process is described. The selected raw material is ultra-high molecular weight polyethylene. The raw fibers are first loosened, and then the raw fibers are combed into a mesh and laminated through the needle punching process to achieve the production of multi-layer flexible anti stab fabrics.

## 4 Movement performance of multi-layer flexible anti stab fabric

The preparation of anti stab fabric is composed of multiple layers of fiber raw materials, and ultra-high molecular weight polyethylene fibers have excellent anti stab performance. However, the prepared multi-layer flexible anti stab fabric ultimately needs to be applied to martial arts, so it is necessary to analyze the sports performance of multi-layer flexible anti stab fabric.

Martial arts requires intense physical activity and coping with sharp devices, so multi-layer flexible anti stab fabrics need to be analyzed for strength and wear resistance. Martial arts athletes need to consider the softness and comfort of multi-layer flexible anti stab fabrics when wearing them for exercise. In addition, martial arts athletes have a large amount of exercise, and their bodies inevitably experience sweating, fever, and other phenomena. It is also important to consider the breathability and moisture permeability of multi-layer flexible anti stab fabrics.

Evaluating the physical and mechanical properties of non-woven materials, woven materials and composite materials is a key step to ensure that the materials can meet the requirements in specific applications. Including evaluating the strength, wear resistance, softness, comfort, air permeability and moisture permeability of materials. The areal densities of the flexible stab-resistant fabric with 2 layers, 3 layers, 4 layers, 5 layers, and 6 layers are 0.2, 0.3, 0.4, 0.5, and 0.6 kg/m^2^ respectively.

### 4.1 Strength and wear resistance

ASTM D5034 standard test method was used to measure the strength and elongation of materials under tensile conditions. The abrasion resistance of materials was measured by ASTM D4157 standard. Prepare samples of high strength polyethylene fiber multilayer flexible stab-resistant fabric composites. According to the experimental requirements and standard requirements, samples with appropriate size and shape are cut out. The tensile strength was tested with a standard tensile tester. Clamp the sample on the tensile tester and record the load and elongation data during the tensile process.

**FIGURE 4:**
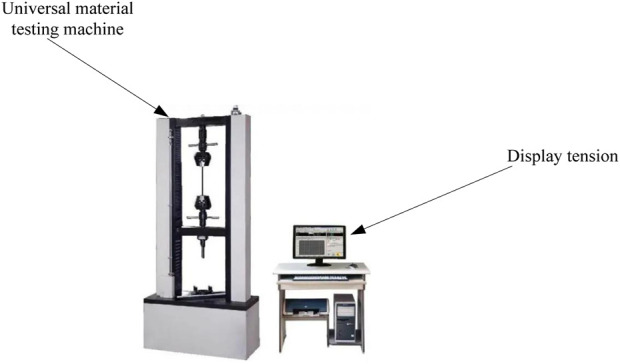
Tensile test scenario.

This article uses ultra-high molecular weight polyethylene fibers as raw materials. The layup structures are: woven fabric, non-woven material, composite material of non-woven material + woven fabric + non-woven material. The number of layers of flexible anti stab fabric is set to 2, 3, 4, 5, and 6 layers. The flexible anti stab fabric sample is cut to a length of 200 mm, and the universal material testing machine is used to conduct the tensile testing of the flexible anti stab fabric. The maximum tensile force of the test sensor is 6kN, and the tensile speed is 40 mm/min. A tension sensor is used to record the breaking strength of each flexible anti stab fabric and the elongation at break of the flexible anti stab fabric. The tension test scenario is shown in Figure 4.

In Figure 4, the tensile test scenario is described, with the main equipment including a universal material testing machine and a computer. The computer is connected to a tensile sensor to display the changes in tensile force.

The process of martial arts can cause fabric friction, and it is necessary to analyze the wear resistance of the fabric. The wear resistance test of multi-layer flexible anti stab fabric refers to repeated friction tests on the fabric. The friction force given in the friction test is 100N, and the friction time is 5 min. The quality changes of the fabric before and after friction are observed. The more the mass of multi-layer flexible anti stab fabric decreases after friction, the poorer the wear resistance of the corresponding multi-layer flexible anti stab fabric. In addition, the wear resistance of the fabric can also be judged from its appearance by observing the damage, shedding, and pilling of the fabric after friction.

### 4.2 Softness and comfort

The application of multi-layer flexible anti stab fabrics in martial arts requires not only basic anti stab functions but also comfortable wearing. Multi-layer flexible anti stab fabric should not affect the normal movement of the human body after wearing, including the normal posture, range, and rhythm of human activities.

The comfort of fabrics can be measured through subjective evaluation methods, combining martial arts athletes, fabrics, and the environment, and analyzing comfort through the subjective evaluation of the wearer. This article randomly selects 90 people for comfort evaluation, and uses predictive average evaluation indicators to evaluate. 90 people are divided into three groups to evaluate the comfort of 4-layer flexible anti stab fabric. The predicted average evaluation indicators are shown in Table 3.

**TABLE 3:** Predicted average evaluation indicators.

Level	Thermal sensation	Value
1	Heat	+3
2	Warm	+2
3	Slightly warm	+1
4	Moderate	0
5	Slightly cool	−1
6	Cool	−2
7	Cold	−3

In Table 3, the predicted average evaluation index is described, which measures the comfort sensation of the human body from the perspective of thermal sensation, and includes a total of 7 levels.

The softness of multi-layer flexible anti stab fabric directly affects the fit between the fabric and the wearer, indicating the fabric’s bending resistance. This article analyzes the softness of multi-layer flexible anti stab fabrics by measuring their comfort angle. A larger comfort angle indicates a more flexible fabric.

### 4.3 Air permeability and moisture permeability

The movement performance of multi-layer flexible anti stab fabric also includes breathability and moisture permeability. Due to raw materials and manufacturing industry reasons, multi-layer flexible anti stab fabric has a high surface density, making it difficult to achieve good breathability and moisture permeability. It is crucial to analyze the breathability and moisture permeability of multi-layer flexible anti stab fabrics.

Fabrics with good breathability can make the wearer more comfortable. GB/T5453-1997 is used to measure the breathability of textile fabrics and compare the breathability of three types of flexible anti stab fabrics. The number of layers and the layer structure of the fabric are the main influencing factors on the breathability of the fabric. Generally, the higher the number of layers of the fabric, the lower the corresponding breathability.

The moisture permeability of a fabric represents its ability to penetrate or absorb the human body’s performance and eliminate steam. The fabric promptly absorbs the sweat emitted by the human body and is discharged into the air. This article uses the cup method to measure the moisture permeability of fabrics, and conducts water vapor permeability tests according to the GB/T1037-1988 standard. The testing area of multi-layer flexible anti stab fabric is set to 1 square meter, and the moisture permeability of multi-layer flexible anti stab fabric is measured within 24 h. The evaluation of fabric moisture permeability is mainly expressed through moisture permeability. The greater the moisture permeability, the better the moisture permeability of the fabric.

### 4.4 Tear strength and cut resistance

Tearing strength refers to the resistance of materials when subjected to tearing force. For flexible stab-resistant fabrics, tear strength directly affects their durability and loss resistance. The stab-resistant fabric with high tear strength means that it has better tear resistance, can withstand external forces and has stronger puncture resistance. Cutting resistance refers to the ability of a material to resist being cut or scratched. The cutting resistance of flexible stab-resistant fabric determines its performance when it is attacked by knives or sharp objects. Good cutting resistance can effectively block potential knife cutting or stabbing, and protect users from injury. The tear resistance of the fabric was measured by ISO 13937-1, and the tear strength of the fabric was measured by ASTM D2261.

## 5 Experimental verification and results

**FIGURE 5:**
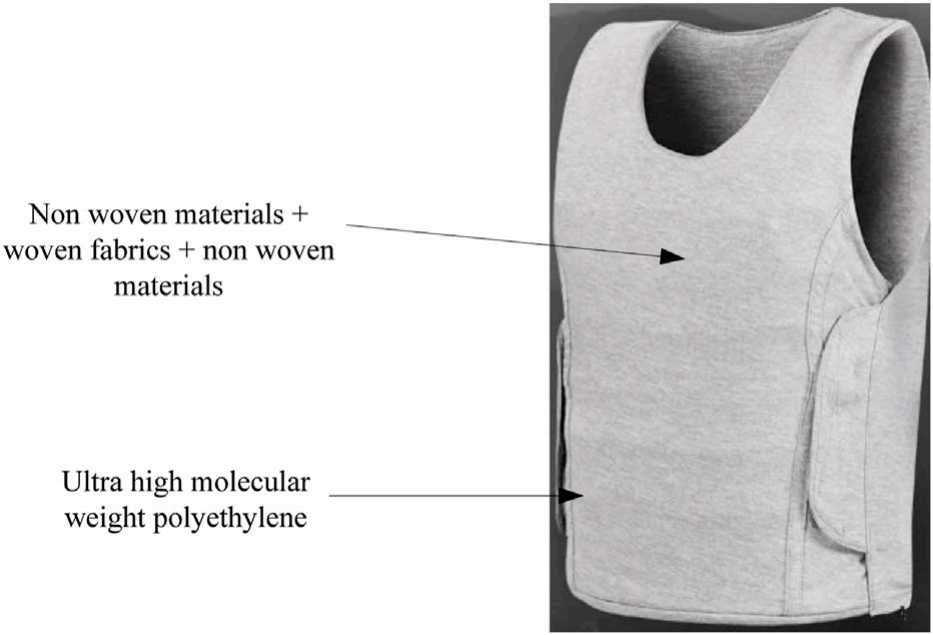
Multi-layer flexible anti stab fabric sample.

This article used ultra-high molecular weight polyethylene fibers as raw materials, with a layer structure of non-woven material + woven fabric + non-woven material. Through pre-treatment, layering, soaking, curing and other steps, multi-layer flexible anti stab fabric was made. The multi-layer flexible anti stab fabric sample is shown in Figure 5.

In Figure 5, a multi-layer flexible anti stab fabric sample is described, which has high strength and can be used in the field of anti stab in martial arts.

### 5.1 Strength and wear resistance experimental results

**FIGURE 6:**
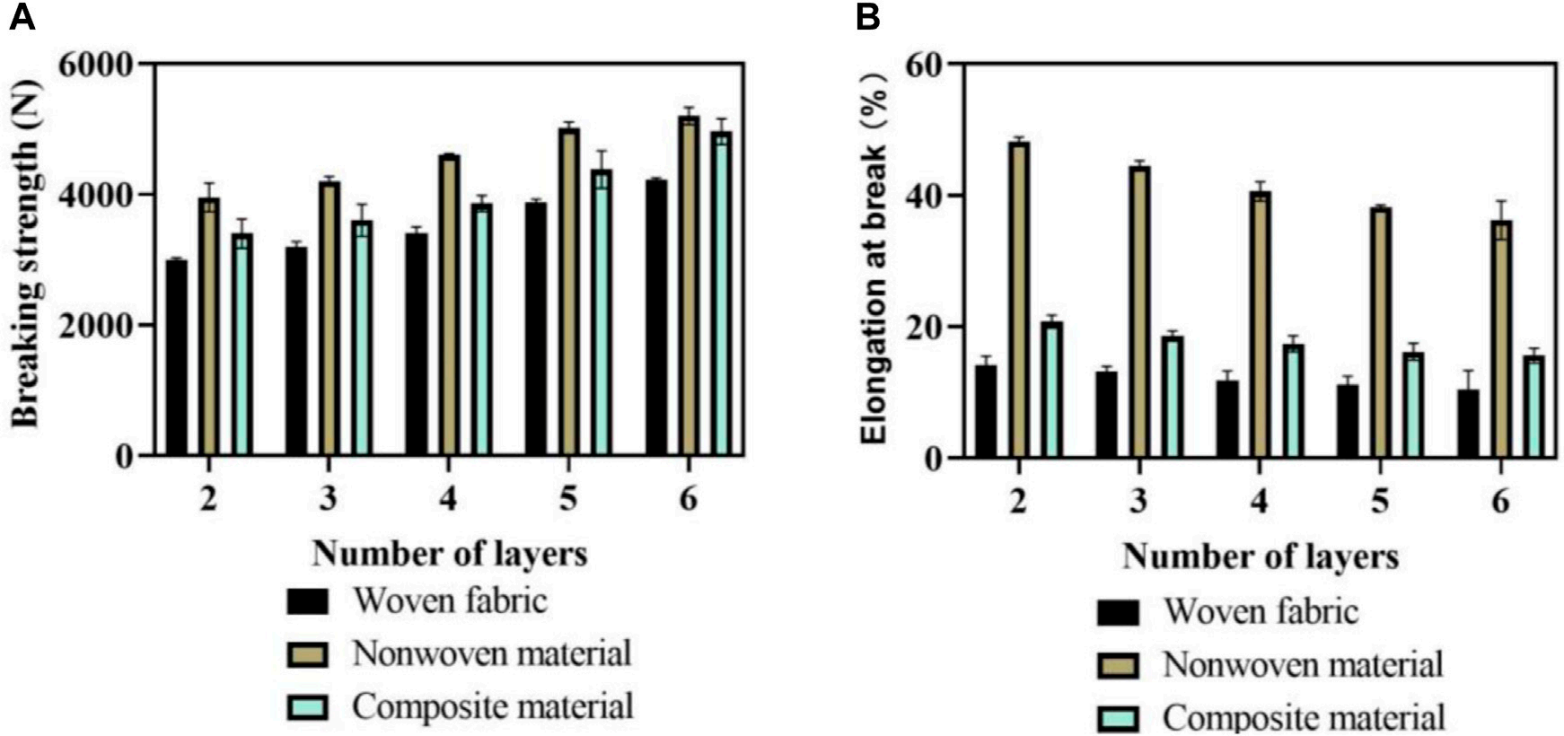
Tensile testing results of fabrics with different layered structures. **(A)** Results of breaking strength. **(B)** Results of elongation at break.

In this paper, tensile testing were carried out on multi-layer flexible anti stab fabrics with three kinds of ply structures. The tensile testing results of fabrics with different ply structures are shown in Figure 6.

In Figure 6A, the fracture strength result is described. The horizontal axis represents the number of layers of flexible anti stab fabric, with 10 sets of tests conducted on each layer. The vertical axis represents the breaking strength. Comparing the multi-layer flexible anti stab fabrics with three layered structures, the breaking force of non-woven materials was greater than that of composite materials, and the breaking force of composite materials was greater than that of woven fabrics. Comparing the breaking force of fabrics with different layers, it can be found that the more layers of the same fabric, the greater the required breaking force. When the number of layers was 4, the average breaking force of woven fabrics was 3400N; the breaking force of non-woven materials was 4600N; and the breaking force of composite materials was 3860N. In Figure 6B, the results of elongation at break are described. The horizontal axis represents the number of layers of flexible anti stab fabric, while the vertical axis represents the elongation at break. Among the three layered structures, the elongation at break of non-woven materials was greater than that of composite materials, while the elongation at break of composite materials was greater than that of woven fabrics. The elongation at break of non-woven materials was very high, mainly due to the fiber arrangement of non-woven materials, which made the overall fabric structure very soft and was stretched very long under tensile force. When the number of layers was 4, the average elongation at break of woven fabrics was 11.8%; the elongation at break of non-woven materials was 40.6%; and the elongation at break of composite materials was 17.4%. Therefore, composite fabrics with a layup structure consisting of non-woven materials, woven fabrics, and non-woven materials can simultaneously have high breaking strength and low elongation at break.

The application of multi-layer flexible anti stab fabric in martial arts requires attention to the wear and tear of the fabric. The analysis results of wear resistance are shown in Table 4.

**TABLE 4:** Analysis results of wear resistance.

Layered structure	Woven fabric	Non-woven material	Composite material
Mass reduction (%)	1.3	1.8	1.1
Damage situation	Less	More	Less
Hair loss situation	Less	More	Less
Pilling situation	Less	More	Less

In Table 4, four layers of flexible anti stab fabric were selected for analysis. By observing the weight reduction of the fabric, the wear resistance of the fabric can be quantitatively observed. The mass reduction of non-woven materials is the most significant, which is closely related to the fiber arrangement of non-woven materials. The mass reduction of non-woven materials was 1.8%; the mass reduction of woven fabrics was 1.3%; and the mass reduction of composite materials is 1.1%. The damage, shedding, and pilling of composite fabrics are also rare. Composite fabrics have excellent wear resistance and can be well applied in the field of martial arts.

### 5.2 Experimental results of softness and comfort

**FIGURE 7:**
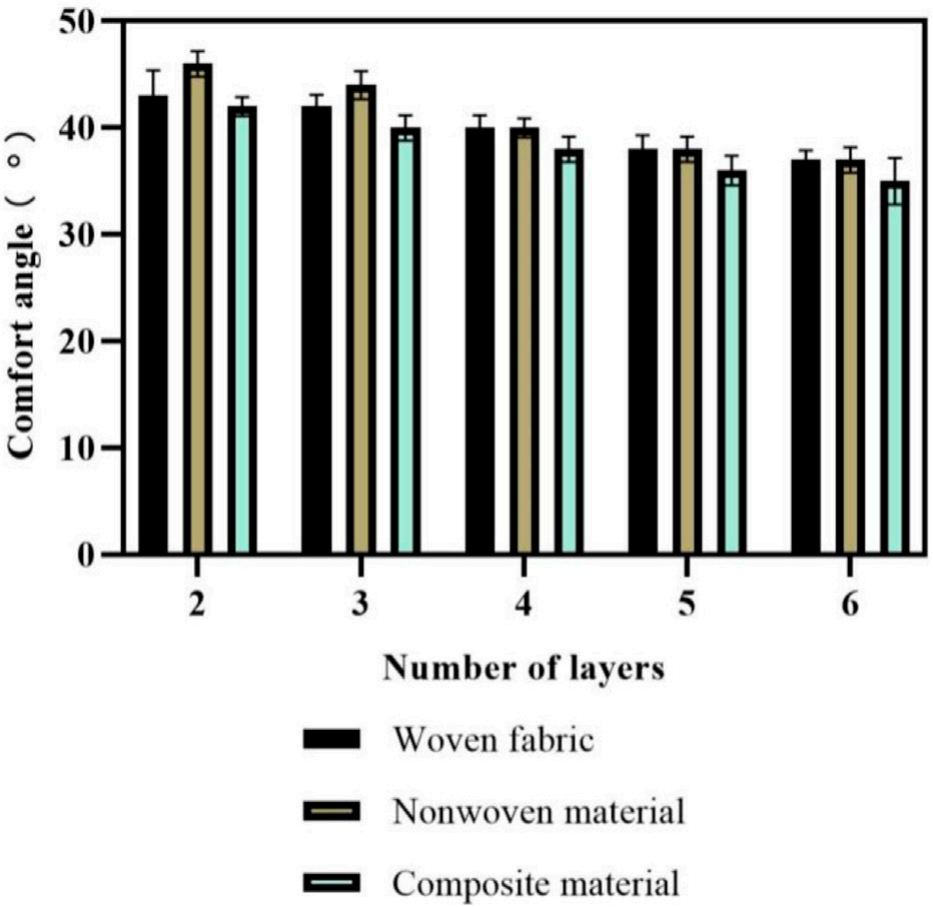
Softness comparison results.

The application of multi-layer flexible anti stab fabric needs to consider the softness of the material. This article compared the softness of fabrics with different layer structures through comfort angles. The softness comparison results are shown in Figure 7.

In Figure 7, the softness comparison results are described. The horizontal axis represents the number of layers of flexible anti stab fabric, with 10 sets of tests conducted on each layer. The vertical axis represents the comfort angle. By comparing the comfort angles of three layered fabrics, the softness of composite fabrics was not very high. In general, the softness of non-woven materials was greater than that of woven fabrics, and the softness of woven fabrics was greater than that of composite materials. When the number of layers was 4, the average comfort angle of woven fabric was 40°; that of non-woven materials was 40°; and that of composite materials was 38°. When the number of layers was 2, the average comfort angle of woven fabric was 43°; that of non-woven materials was 46°; and that of composite materials was 42°. Therefore, the softness performance of composite fabrics is slightly inferior to the other two materials, which leads to a harder texture of complex material fabrics.

The application of multi-layer flexible anti stab fabric in martial arts requires an analysis of the comfort of wearing. Comfort is a subjective concept that is related to factors such as fabric weight, thickness, breathability, and moisture permeability. This article evaluated comfort by predicting average evaluation indicators. The predicted average evaluation values are shown in Table 5.

**TABLE 5:** Predicted average evaluation values.

Layered structure	Value	Thermal sensation
Woven fabric	+1	Slightly warm
Non-woven material	+1	Slightly warm
Composite material	0	Moderate

In Table 5, the predicted average evaluation values are described. The tester’s thermal perception of composite fabrics is moderate, indicating that they are very comfortable to wear. The predicted average evaluation values for both woven and non-woven materials are +1, and the thermal sensation of the testers is warm. The wearing of woven and non-woven materials is relatively warm, which may be related to the weight and thickness of the fabric. Therefore, the wearing comfort of complex material fabrics is high, providing a comfortable dressing environment for martial arts athletes.

### 5.3 Air permeability and moisture permeability test results

**FIGURE 8:**
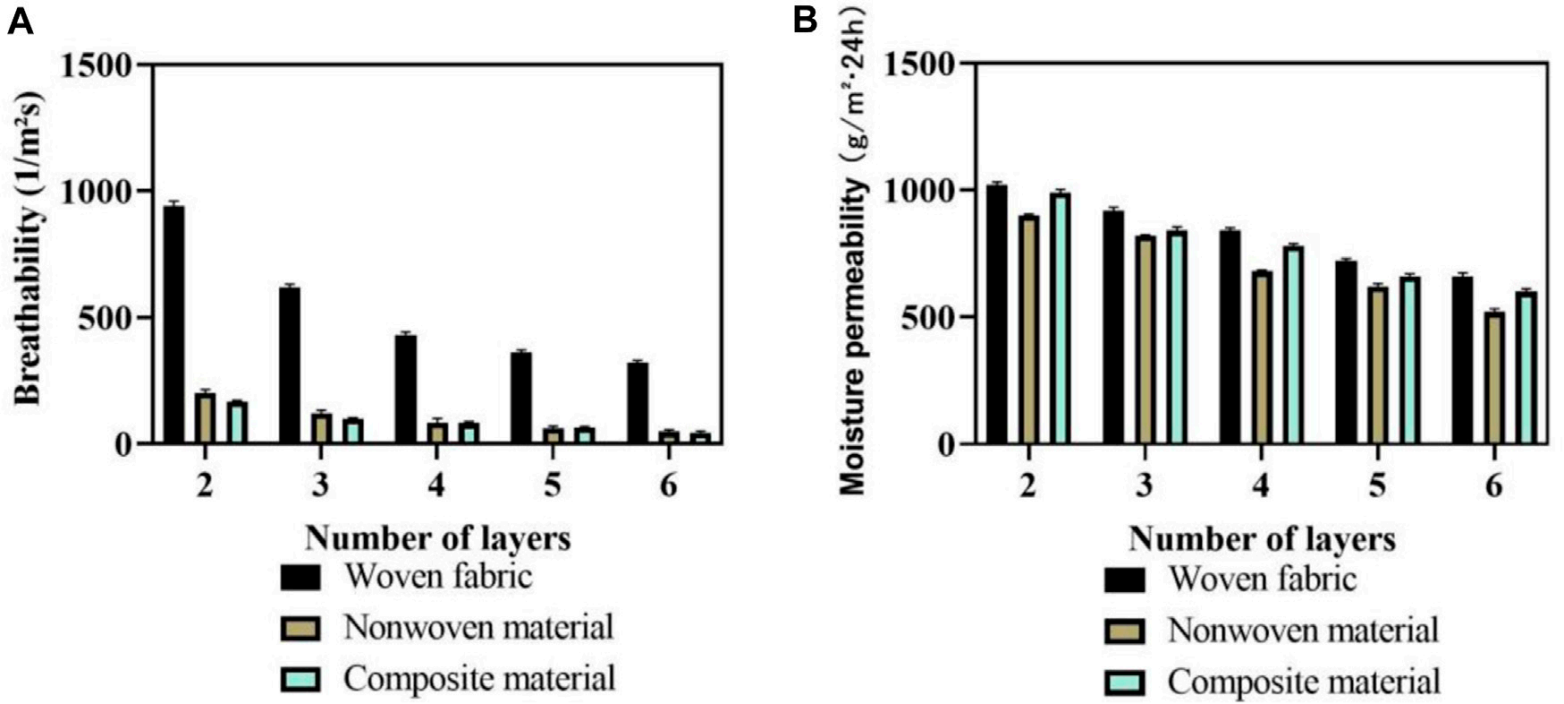
Analysis results of air permeability and moisture permeability. **(A)** Permeability results. **(B)** Moisture permeability results.

The multi-layer flexible anti stab fabric in martial arts aims to provide a safe anti stab environment and excellent athletic performance for martial arts athletes. This article analyzed the breathability and moisture permeability of fabrics with different layer structures. The analysis results of air permeability and moisture permeability are shown in Figure 8.

In Figure 8A, the results of fabric breathability are described. The horizontal axis represents the number of layers of flexible anti stab fabric, while the vertical axis represents breathability. By comparing the breathability of three types of fabrics, the breathability of woven fabrics was greater than that of non-woven materials, and the overall breathability of non-woven materials was greater than that of composite materials. The breathability of all three fabrics decreases with the increase of the number of layers, and the breathability of composite fabrics still needs to be improved. When the number of layers was 4, the average permeability of woven fabric was 430 L/m^2^s; the average permeability of non-woven materials was 84 L/m^2^s; and the average permeability of composite materials was 82 L/m^2^s. When the number of layers was 6, the average permeability of woven fabric was 320 L/m^2^s; the average permeability of non-woven materials was 48 L/m^2^s; and the average permeability of composite materials was 44 L/m^2^s. The permeability of composite materials was relatively poor, mainly due to the needle punching process bringing fibers into the warp and weft yarns, resulting in tighter gaps between fabrics. In Figure 8B, the moisture permeability results are described. The vertical axis represents the moisture permeability of 1 square meter of fabric in a single day. Comparing the moisture permeability of three layered fabrics, it can be found that the moisture permeability of woven fabrics was greater than that of composite materials, and the moisture permeability of composite materials was greater than that of non-woven materials. When the number of layers was 4, the average moisture permeability of woven fabrics was 840 g/m^2^ ∙ 24 h; that of non-woven materials was 680 g/m^2^ ∙ 24 h; and that of composite materials was 780 g/m^2^ ∙ 24 h. When the number of layers was 6, the average moisture permeability of woven fabrics was 660 g/m^2^ ∙ 24 h; that of non-woven materials was 520 g/m^2^ ∙ 24 h; and that of composite materials was 600 g/m^2^ ∙ 24 h. Therefore, the breathability and moisture permeability of composite fabrics have not yet reached a high level, and woven fabrics have excellent breathability and moisture permeability.

### 5.4 Tear strength and cut resistance results

**FIGURE 9:**
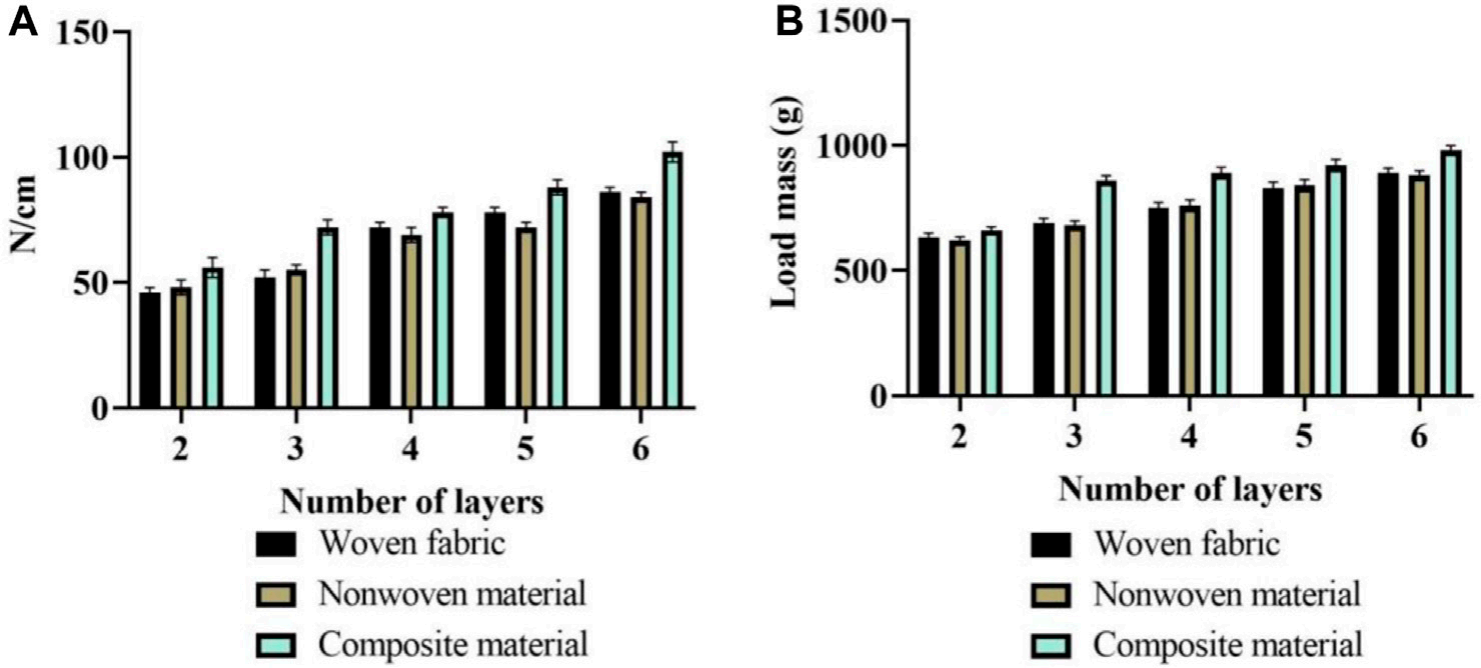
Tear strength and cut resistance. **(A)** Tear strength. **(B)** Cut resistance.

The comparison results of tear strength and cut resistance of fabrics with different ply structures are shown in Figure 9.

In Figure 9A, the tear strength of fabrics with different ply structures is described. It can be seen that the tear strength of composite fabrics is higher than that of the other two ply structures, and the more layers of fabrics, the greater the tear strength. Higher tear strength means that the stab-resistant fabric has better tear resistance. In Figure 9B, the cutting resistance of fabrics with different ply structures is described. The cutting resistance of composite fabrics is higher than that of the other two ply structures. The higher cutting resistance indicates that stab-resistant fabrics have better resistance to cutting or scratching.

### 5.5 Stab-proof performance analysis

**FIGURE 10:**
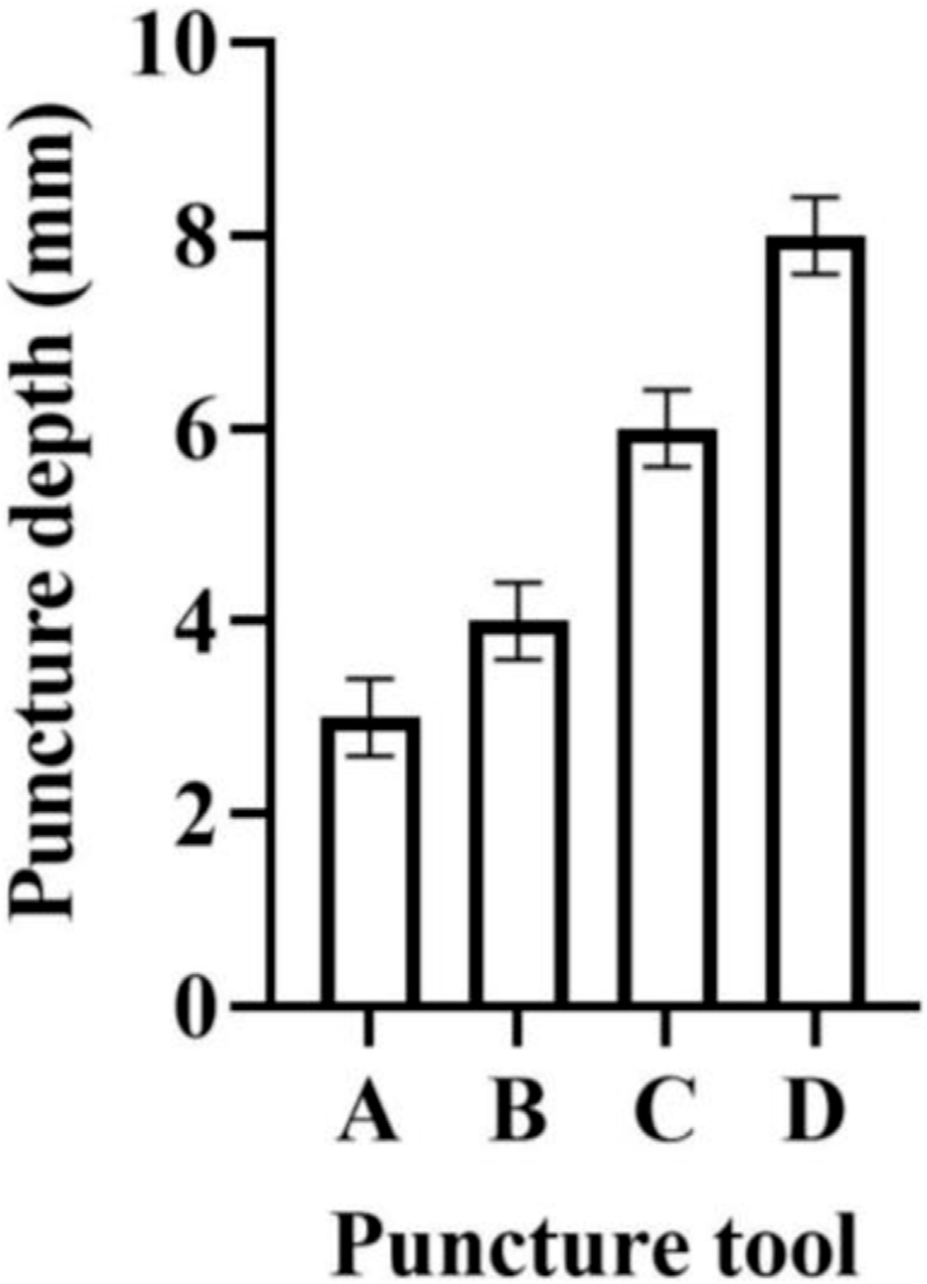
Stab resistance.

The stab resistance test was carried out with sharp metal blocks, glass fragments, blades and needles. The stab strength was 100N, the puncture speed was 1 m/s, and the puncture depth was recorded. The stab resistance of the 6-layer composite fabric is shown in Figure 10.

In Figure 10, the stab-resistant performance of a six-layer composite fabric is described, and A, B, C and D respectively represent sharp metal blocks, glass fragments, blades and needles. The penetration depth in the metal block is the lowest, and the penetration depth using the needle tool is the deepest. Under the test of four kinds of puncture objects, the puncture depth of 6-layer composite fabric is less than 1 cm.

## 6 Conclusion

With the integration of the global economy, people are paying more and more attention to the development of martial arts. Martial arts can strengthen the body, but there is also a threat of sharp devices such as knives and swords in the process of martial arts. It is very important to prepare fabrics with anti stab performance. This article used ultra-high molecular weight polyethylene fibers as raw materials to produce multi-layer flexible anti stab fabrics through pre-treatment, layering, wetting, curing, and other steps. This article achieved the preparation of composite materials consisting of non-woven materials, woven fabrics, and non-woven materials through needle punching technology, and compared the motion performance of three types of layered fabrics. This article evaluated the sports performance of multi-layer flexible anti stab fabrics, including strength and wear resistance, softness and comfort, breathability and moisture permeability. The experimental results showed that the composite material using non-woven materials, woven fabrics, and non-woven materials can simultaneously have high breaking strength and low elongation at break, and the wearing comfort of the composite material was high. However, the overall breathability and moisture permeability of the composite material are not optimal. The multi-layer flexible anti stab composite fabric prepared in this article has excellent wear resistance by laminating fibers, which can be well applied in the field of martial arts. However, it still needs to improve the breathability of the fabric. This article did not analyze the weight of multi-layer flexible anti stab fabric when analyzing its motion performance. Therefore, analyzing the weight of fabrics with different layer structures would be the direction of future research.

## Data Availability

The original contributions presented in the study are included in the article/Supplementary Material, further inquiries can be directed to the corresponding author.
